# Disseminated fusariosis in children: Report of two cases in girls with leukemia

**DOI:** 10.18502/cmm.8.1.9213

**Published:** 2022-03

**Authors:** Alixandra De la Espriella, Andrea Restrepo, Mónica Trujillo, Karen Arango

**Affiliations:** 1 CES University, Medellin, Colombia; 2 Pablo Tobón Uribe Hospital, Medellín, Colombia; 3 Corporation for Center for Biological Research, Pontifical Bolivarian University, Medellin, Antioquia, Colombia

**Keywords:** *Fusarium* spp., Invasive fungal infection, Leukemia, Neutropenia

## Abstract

**Background and Purpose:**

Disseminated fusariosis is an opportunistic infection caused by the hyaline fungus *Fusarium* spp. and occurs mainly in patients with leukemia.

**Case report:**

Two cases of disseminated fusariosis in pediatric patients are presented. Profound and prolonged neutropenia, fever, myalgia, and skin lesions in the legs were present in two girls with leukemia undergoing chemotherapy. In the first case, infection by *Fusarium* spp. was confirmed by anatomopathological findings, pathogen isolation, and polymerase chain reaction. In the second case, *Fusarium solani* infection was confirmed by mass spectrometry using blood cultures and skin lesion samples.

**Conclusion:**

It is important to consider disseminated fusariosis in high-risk patients who present with profound and prolonged neutropenia and persistent fever that does not resolve after broad-spectrum antibiotics to initiate antifungal therapy in a timely manner.

## Introduction

Disseminated fusariosis is observed in patients with acute leukemia (70%-80%) who present with profound and prolonged neutropenia [ 1 ]. This pathogen enters the organism through inhalation, ingestion, or direct inoculation. The localized disease in immunocompetent persons or disseminated disease in immunosuppressed persons manifests this infection [ 2 ]. Resistance of *Fusarium* spp. to most antifungals makes treatment difficult. It is frequently observed that clinical response is discordant with in vitro behavior, which reflects the versatility of this pathogen as its main characteristic [ 3 ].

## Case report

### Case no. 1

**Figure 1:**
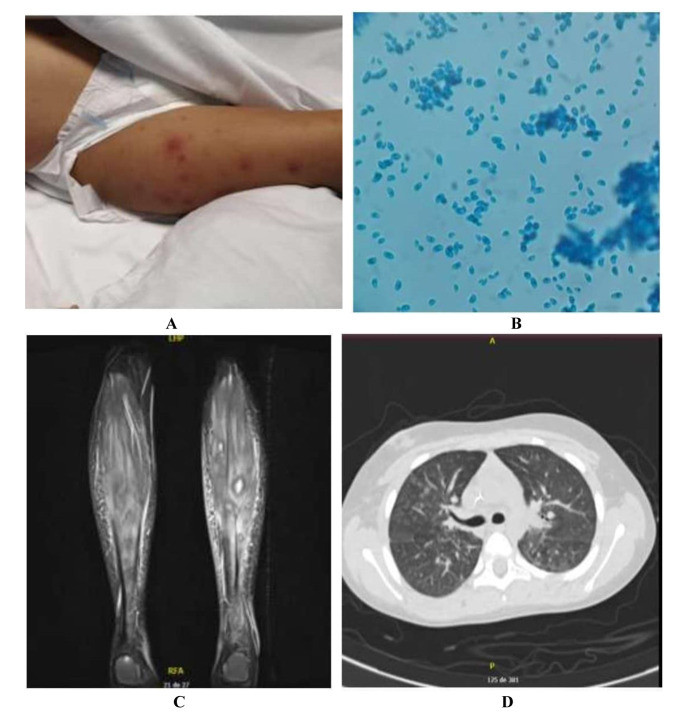
Some results of case 1. A = skin lesions; B = direct examination of the culture, staining with lactophenol blue: phialides, sickle-shaped micro and macroconidia; C = magnetic resonance of the lower limb sagittal section. D = chest computerized axial tomography: patched micro areas with ground glass opacity in all lobes with upper right predominance and apical segment of both lower lobes.

A 10-year-old girl with high-risk B-cell acute lymphoblastic leukemia who relapsed with 91% lymphoblasts underwent salvage chemotherapy through a totally implantable venous access port. During the hospital stay, she presented two episodes of febrile neutropenia, the first without microbiological isolation and the second with neutropenic colitis and extended-spectrum beta-lactamase producing *Escherichia coli* (*E. coli*) bacteremia with secondary septic shock and an episode of oral herpes simplex. The patient received several broad-spectrum antibiotic regimens, including meropenem, vancomycin, cefepime, and prophylaxis for the underlying disease with voriconazole (6 mg/kg/day), acyclovir (30 mg/kg/day), and trimethoprim sulfamethoxazole (5 mg/kg/day) every other day. On day 22 of the hospital stay, the patient presented with a fever of 39.5 degrees Celsius, erythematous violaceous papules with a necrotic center in the skin (Figure 1A), limitation of gait, and intense pain on palpation of the gastrocnemius muscles. The case had neutropenia (neutrophils=0/mm^3^) for 17 days and was treated with vancomycin and meropenem in dosages of 60 mg/kg/day and 100 mg/kg/day, respectively. However, due to the patient’s underlying disease and associated risk factors, invasive fungal infection was suspected, empirical therapy was initiated with liposomal amphotericin B (5 mg/kg/day), and prophylactic voriconazole was stopped. In the skin biopsy, septate hyaline hyphae were observed in the mid-dermis and the lumen of some vessels. Additionally, in the Becton-Dickinson cultures of this sample, mold growth was observed, whose microscopic characteristics revealed a crescent-shaped micro and macroconidia emerging from a specialized cell type phialide as typical characteristics of *Fusarium* species (Figure 1B). In the magnetic resonance imaging (MRI) of the lower limbs (Figure 1C), there were intramuscular and fascial microabscesses, accompanied by acute myositis, and bone microabscesses in the bilateral distal and proximal meta-epiphyseal regions of the tibia and fibula. The panfungal polymerase chain reaction (PCR) was conducted accompanied with sequencing, in which the D1D2 region of ribosomal DNA was amplified with primers NL1 and NL4 of skin lesions, and peripheral vein and catheter (port) blood cultures were positive for *Fusarium* spp. Subsequently, the port was removed and the patient presented partial clinical improvement. Moreover, although the pain in the gastrocnemius decreased, the fever persisted despite the recovery of the neutrophil count (Table 1).

**Table 1:** Blood chemistry evolution, clinical case number 1.

Parameter	Days of disease progression					
	Day 1	Day 5	Day 6 †	Day 22 *	Day 33 **	Day 43 ***
Hemoglobin (g/l)	13	12.5	10.8	5.6	7.1	
Hematocrit (%)	36	34.8	30.2	16.2	20.5	
Leukocytes (mm^3^)	4000	3800	1000	0	3100	
Neutrophils% (mm^3^)	47% (1880)	36% (1368)	58% (580)	0	89% (2759)	
Lymphocytes% (mm^3^)	46% (840)	40.7% (1550)	33% (330)	0	3% (93)	
Platelets (mm^3^)	30,000	26,000	290,000	15,000	50,000	
Creatinine (mg/dl)				0.36		1.29
Urea nitrogen (mg/dl)				17.9		21.2
Sodium (mmol/l)				135		134
Potassium (mmol/l)				4.9		4.4
Calcium (mmol/l)				8.6		9.3
Chlorine (mmol/l)				100		100
CPK (mmol/l)				38		
AST (U/L)				13		
ALT (U/L)				17		

† 24 hours post chemotherapy

* onset of fever, skin lesions and pain in the lower extremities.

** recovery of neutrophils, persistence of fever, initiation of salvage therapy with voriconazole.

*** corresponds to day 21 of treatment with liposomal amphotericin B.

Therefore, it was decided to add intravenous voriconazole in a dosage of 16 mg/kg/day on day 11 of therapy with liposomal amphotericin B. Eventually, the fever subsided two days after the start of voriconazole and 10 days after the start of liposomal amphotericin B.

Susceptibility tests were interpreted as intermediate or dose-dependent susceptibility to voriconazole (minimum inhibitory concentration (MIC): 3 µg/mL) and resistance to amphotericin B (MIC: 8 µg/mL). In addition, a search was performed using images of possible distant seeding foci with computerized axial tomography (CAT) of the paranasal sinuses, which was normal, as well as chest CAT that showed areas with ground-glass opacity (Figure 1D). In total, the patient received amphotericin B for 21 days, which was suspended due to renal failure (creatinine 1.29 mg/dL) and intravenous voriconazole for 14 days. The administration of oral voriconazole was continued since another cycle of consolidation chemotherapy was performed to achieve optimal serum levels of 2 mg/L. The patient had a satisfactory clinical evaluation with the disappearance of fever and skin lesions. The patient died four months later due to a new relapse of leukemia.

### Case no. 2

A 3-year-old girl with a diagnosis of acute myeloid leukemia with differentiation to neutrophils was undergoing treatment with induction chemotherapy through a totally implantable venous access port. On day 10 of chemotherapy, she presented abdominal pain, liquid stools, fever of 39 degrees Celsius, heart rate of 110 beats per minute, respiratory rate of 20 breaths per minute, blood pressure of 78/48 mmHg, and pain on palpation of the abdomen with neutrophil count 0/mm^3^ (Table 2). *E. coli* was isolated from the blood cultures of the patient. She was treated with piperacillin/tazobactam (400 mg/kg/day) for 10 days. In addition, she received prophylaxis for her underlying disease with trimethoprim sulfamethoxazole (5 mg/kg/day) three times a week, voriconazole (16 mg/kg/day) every 12 h, and acyclovir (30 mg/kg/day) every 8 h. Despite the improvement of abdominal pain and diarrhea, fever persisted, and new blood cultures showed growth of budding blastoconidia. Caspofungin was started at 50 mg/m^2^/day after a loading dose of 70 mg/m^2^/day. Extension studies were carried out to look for distant seedings of the fungus: abdominal ultrasound and normal echocardiography, reviewed by the ophthalmology service who reports fundus without lesions. Seven days later, the patient presented generalized erythematous maculopapular skin lesions with a violaceous center associated with pain in the gastrocnemius. A biopsy of the skin lesions showed septate hyaline hyphae. The growth of white, flat, and fuzzy colonies compatible with *Fusarium* spp. was observed on peripheral vein blood culture. Spindle or crescent-shaped phialides with macro and microconidia were observed through microscopic preparations with lactophenol blue made from the culture. Growth of *Fusarium solani* in blood cultures was identified by matrix-assisted laser desorption/ionization time-of-flight (MALDI-TOF) mass spectrometry (equipment Bruker, of the company Becton Dickinson with BDLA library, New Jersey, USA) (Table 3). Growth of *Fusarium* spp. was observed in peripheral vein and catheter control blood cultures. Therefore, the catheter was removed, and negative blood cultures were achieved subsequently. Liposomal amphotericin B (5 mg/kg/day) was started, caspofungin was suspended, and voriconazole was continued. Antifungal sensitivity tests showed elevated MICs (Table 3).

**Table 2:** Blood chemistry evolution, clinical case number 2

Days of disease progression	Day 1	Day 4	Day 8
Hemoglobin (g/l)	8.9	7.8	8.9
Hematocrit (%)	25.3	22.2	25.9
Leukocytes (mm^3^)	400	400	400
Neutrophils (mm^3^)	0	0	1
Lymphocytes (mm^3^)	3	4	2
Platelets (mm^3^)	21,000	33,000	33,000

**Table 3:** Microbiology clinical case number 2

Test	Results
Peripheral blood cultures in aerobic medium: mass spectrometry (MALDI-TOF)	*Fusarium solani*
Skin lesions: MALDI-TOF	*Fusarium solani*
Sensitivity tests	Posaconazole: 32
MIC in micrograms/mL,	Amphotericin B: 3
Based on document CLSI M38-A2.	Voriconazole > 32
Diffusion technique with E-TEST strips	
RT PCR SARS COV2 nasopharyngeal swab	Positive
Serum levels of voriconazole	1.6 mg/L (valor normal: 4 -6 mg/L)

MIC: minimum inhibitory concentration, data based on in vitro reports obtained with many isolates.

Chest-abdominal CT showed no pathological findings; however, in the lower limb ultrasound, the gastrocnemius microabscesses without liquefaction were observed which was approximately 9.6 x 5.5 mm in diameter and not susceptible to percutaneous drainage. On the eighth day of treatment with liposomal amphotericin B, the patient presented an episode of desaturation, respiratory distress, and increased oxygen requirements. The chest radiograph showed perihilar interstitial opacities with laminar atelectasis and neutropenic persistence (neutrophils: 4/mm^3^). Due to the COVID-19 pandemic, a reverse transcriptase-polymerase chain reaction was performed for SARS COV 2, which was positive.

Despite having recovered from neutropenia and having negative control blood cultures, the patient remained febrile. At 30 days of treatment with liposomal amphotericin B, pain and edema of the left elbow appeared. The MRI of the affected joint indicated signs of osteoarthritis which required surgical drainage, and no microbiological growth was observed in the obtained cloudy fluid. A few days later, the patent presented subcutaneous nodules in the lower limbs, edema, and pain in both ankles and the second finger of the left hand, without joint effusion, with the persistence of fever. Total body MRI indicated acute multifocal disseminated osteomyelitis with extensive involvement of the appendicular skeleton, associated with myositis and microabscesses not susceptible to drainage in both subcutaneous and intramuscular cellular tissue, in upper and lower extremities, and septic arthritis of both elbows. Possibly all this was due to the spread of infection caused by *Fusarium solani*. The patient required another surgical intervention, during which multiple encapsulated abscesses were found. The largest multiple encapsulated abscess was drained, and multiple samples of bone, tissue, and joint fluid were sent to the microbiology laboratory, which was seeded in culture media for aerobes, mycobacteria, and fungi. However, no growth was obtained.

A small cylindrical structure observed in the tissue biopsy could correspond to a fragment of hyphae and multiple abscessed epithelioid granulomas. It was decided to add caspofungin (50 milligrams/m^2^/day) after a loading dose of 70 mg/m^2^/day to obtain synergism with the other antifungals. In total the patient received 6 months of treatment with Amphotericin B, and voriconazole in addition to 4 months of caspofungin due to bone involvement, which resulted in resolution of the skin and bone lesions. The total body MRI after 6 months showed resolution of almost all lesions and only residual inflammation persisted in the calcaneus and left fibula. At the time this case was written, the patient was in remission and received maintenance chemotherapy. In addition, voriconazole was continued as secondary prophylaxis.

## Discussion

The prevalence of opportunistic infections caused by *Fusarium* spp. has increased in patients immune-compromised by immunosuppressive treatments [ 3 , 4 ]. The entrance into the body is through inhalation and ingestion causes sinusitis and pneumonia and mycotoxicosis, respectively. Moreover, traumatic inoculation is associated with foreign bodies, such as contact lenses and vascular devices [ 1 , 2 ]. Skin and nail lesions are important entry points. The risk of developing disseminated fusariosis from infected nails is high in patients with cancer in the profound neutropenic phase [ 5 ]. 

Fusariosis mortality is high (80%) due to high resistance of *Fusarium* spp. to multiple antifungals and extensive necrosis of the tissues with dissemination to multiple organs which can lead to immunosuppression in the host [ 3 ]. *Fusarium solani* is the complex most often found in clinical isolates (40-60%). It is considered to be the most virulent complex supported by assays in murine models [ 6 ]. The disseminated disease generally occurs in patients with profound and prolonged neutropenia and manifests with persistent fever, myalgia, skin lesions, and fungemia, with or without compromise of other organs [ 2 , 4 , 7 ]. 

The diagnosis should be conducted through the isolation of the fungus from the blood, biopsy of skin lesions, or other affected anatomical sites. Blood cultures can be positive in up to 50% of cases. However, diagnosis remains difficult due to the lack of sensitivity of diagnostic tests in patients with hematological pathologies who receive prophylaxis for molds [ 1 , 8 ]. Identification of species is performed through panfungal PCR assay with sequencing or by mass spectrometry [ 2 , 9 ]. For the identification of the genus Fusarium from the culture, it is necessary to observe phialides with spindle-shaped micro-and macroconidia (in the form of “crescent” or “banana”), which may vary in shape and size, depending on the isolated species. In tissues, it is only possible to observe hyaline septate hyphae, which may be branched and form acute angles, similar to those observed in infections caused by Aspergillus species and other hyalohyphomycoses [ 10 ].

The treatment has three pillars, including antifungal therapy, recovery of immunity, and surgery. The efficacy of the treatment is not well established due to the lack of clinical trials and the low frequency of the disease, especially in children. Accordingly, it was important to present the two clinical cases, the heterogeneity of the infection, and the underlying condition of the patients. Combined antifungal therapies represent attractive options, but their benefit is difficult to demonstrate. For all the above, the optimal treatment strategy for severe Fusarium infection continues to be a challenge for clinicians. 

Based on the review of the literature on the adult patients, that has been extrapolated to pediatric patients, most patients (80%) have been treated with combined therapy, surgery (30%), and immunomodulatory therapy with a granulocyte-colony stimulating factor or granulocyte transfusion (40%). A survival rate of only 50% of the reported cases has been obtained through the adoption of these therapies. In some reports, posaconazole or isavuconazole has been used as salvage therapy, with a survival rate of 44% [ 9 ]. The first-line treatment of choice in fusariosis is liposomal amphotericin B. The U.S. Food and Drug Administration approved the use of voriconazole as a second-line drug, and the recommended serum levels to be reached are extrapolated from the treatment for aspergillosis (1-5 mcg/ml) [ 1 ]. The duration of treatment should be individualized based on the site, extent of infection, and immunological status of the patient. The restoration of immune status is essential for a successful therapeutic outcome [ 9 ]. To this purpose, high-risk patients should be identified and ensure that: 1) they are hospitalized in rooms with high-efficiency particulate air filters and positive pressure, 2) the water taps have filters and remain clean, 3) skin and nail lesions are treated before initiating chemotherapy, and 4) adequate antifungal prophylaxis is administered [ 2 ].

## Conclusion

Fusariosis is a disease with high mortality, mainly in high-risk patients. Early recognition of this entity through the confirmation of patients’ signs and symptoms by laboratory methods is essential for the establishment of appropriate and timely antifungal therapy.

The two cases reported in this manuscript were a great treatment challenge to the researchers; however, the objectives were achieved and the patients survived.

The report of these cases can help contribute knowledge to the medical community.

## Authors’ contribution

A. D. L. E. developed the concept, participated in data collection and analysis, performed the literature review, and wrote the first draft of the manuscript. A. R. contributed to the initial idea of the study, participated in data analysis, and reviewed the final version. M. T. contributed to the initial idea of the study, data analysis, and review of the final version. K. A. participated in the literature review and review of the final version.

## Financial disclosure

The authors did not receive any financial support for this study.

## Ethical Considerations

Informed consent was obtained from the patients’ guardians. This study was approved by the Human Ethics Committee of CES University, Medellin, Colombia (Code No. FR-IN-024).

## Acknowledgments

The authors would like to acknowledge Dra. Ana Cristina Ruiz from Pablo Tobón Uribe Hospital, Medellín, Colombia, for histopathological examination of the patients’ skin.

## Conflicts of interest

The authors declare that they have no conflicts of interest regarding the publication of the present study.
